# Ten tips on how to deal with calciphylaxis patients

**DOI:** 10.1093/ckj/sfaf098

**Published:** 2025-04-09

**Authors:** Api Chewcharat, Sagar U Nigwekar

**Affiliations:** Division of Nephrology, Department of Medicine, Brigham and Women's Hospital, Boston, MA, USA; Division of Nephrology, Department of Medicine, Massachusetts General Hospital, Boston, MA, USA

**Keywords:** calciphylaxis, diagnosis, sodium thiosulfate, vitamin K, warfarin

## Abstract

Calciphylaxis is a devastating disease characterized by painful ischemic necrotic skin lesions, primarily affecting patients with end-stage kidney disease. Its incidence has significantly increased over the past decade. Despite substantial morbidity and mortality, the pathogenesis of calciphylaxis remains poorly understood, and currently there are currently no approved treatments available. Diagnosis largely relies on clinical suspicion; skin biopsy may be useful but is not essential, particularly when biopsy risks outweigh benefits. Effective management requires a multidisciplinary approach. Current therapeutic strategies include controlling metabolic bone disease, substituting vitamin K antagonists with alternative anticoagulants, discontinuing calcium and vitamin D supplements, optimizing dialysis protocols, and considering sodium thiosulfate. Promising novel therapies are now emerging. These 10 tips aim to help clinicians avoid common pitfalls and deliver optimal care to patients with calciphylaxis.

## INTRODUCTION

**Figure 1:**
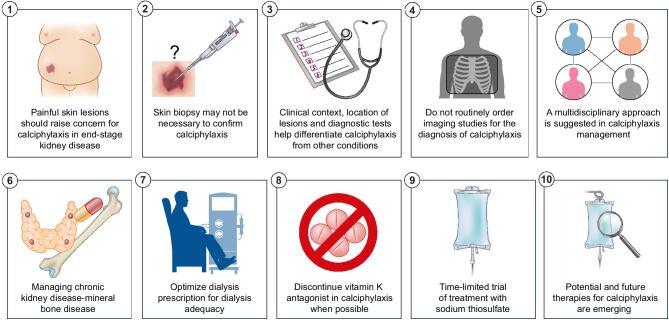
Ten tips on how to deal with calciphylaxis patients.

Calciphylaxis is a devastating disease characterized by excruciatingly painful ischemic necrotic lesions predominantly affecting patients with end-stage kidney disease. Although calciphylaxis was historically considered rare, the incidence of calciphylaxis has been increasing over the past decade from 0.05–0.1 to 3.5–5 new cases per 1000 chronic hemodialysis patient-years [1, 2]. Most calciphylaxis cases were reported from high-income countries and Western countries, likely reflecting both better access to diagnostics tools and higher prevalence of certain calciphylaxis risk factors such as obesity and diabetes. In contrast, data from Japan indicated a significantly lower incidence of calciphylaxis below 0.1 new cases per 1000 chronic hemodialysis patient-years. However, a national survey showed that 60% of Japanese nephrologists did not know the disease itself, suggesting that under-recognition contributed to the low reported rates [3]. Even though calciphylaxis is associated with significant morbidity and mortality, the pathogenesis remains poorly understood, and there are currently no approved treatments. Clinicians should maintain a high index of suspicion for patients with advanced kidney disease presenting with painful skin lesions. While a skin biopsy can help rule out the disease mimics, it is not mandatory when the clinical presentation strongly suggests calciphylaxis, especially when the biopsy carries risks of poor wound healing and secondary infection such as in patients who are on immunosuppressive therapy for autoimmune diseases or transplantation or those with compromised perfusion and malnutrition. Management of calciphylaxis requires a multidisciplinary team, including dermatology, nephrology, nutrition, pain and palliative medicine, wound care and therapist. Key aspects of addressing calciphylaxis involve controlling metabolic bone disease parameters, switching vitamin K antagonists to alternative anticoagulants, stopping calcium supplements and vitamin D analogs, optimizing dialysis prescription, and off-label use of sodium thiosulfate. Several novel and experimental therapies for calciphylaxis are on the horizon. Here, based on clinical experience and published literature, we outline 10 tips for the management of calciphylaxis (Fig. 1).

## TIP 1. PATIENTS WITH ADVANCED CHRONIC KIDNEY DISEASE OR END-STAGE KIDNEY DISEASE PRESENTING WITH PAINFUL SKIN LESIONS SHOULD RAISE CONCERN FOR CALCIPHYLAXIS

Calciphylaxis should be considered in patients with advanced chronic kidney disease (CKD) or end-stage kidney disease (ESKD) who present with painful retiform purpura, subcutaneous nodules or plaques, nonhealing ulcers or cutaneous necrosis, particularly in central body areas with high adiposity, such as the abdomen, buttocks and thighs. While calciphylaxis predominantly occurs among CKD and ESKD patients, it may also develop in non-CKD and kidney transplant patients [4].

Patients with calciphylaxis have considerable mortality rate of 37% at 6 months [5–7]. Risk factors for calciphylaxis include female sex, obesity, diabetes, inflammatory and autoimmune conditions, recurrent skin trauma, medications such as warfarin, iron, calcium and vitamin D analogs, abnormal metabolic bone disease parameters (hypercalcemia, hyperphosphatemia, hyperparathyroidism or over-suppressed parathyroid hormone with adynamic bone disease) and a hypercoagulable state [4, 8].

## TIP 2. SKIN BIOPSY FOR DIAGNOSTIC CONFIRMATION OF CALCIPHYLAXIS MAY NOT BE NECESSARY AMONG ESKD PATIENTS PRESENTING WITH CLASSIC LESIONS OF CALCIPHYLAXIS

**Figure 2:**
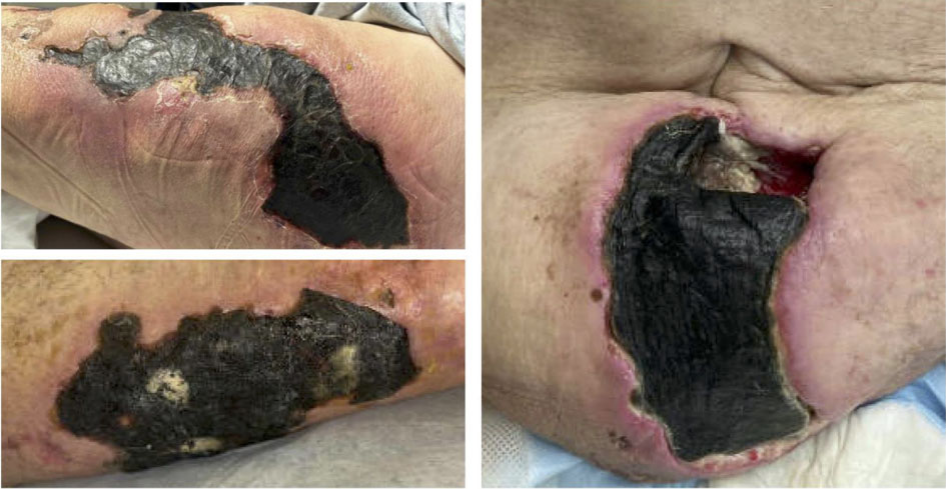
Skin lesions.

Calciphylaxis is characterized by excruciatingly painful ischemic necrotic lesions [9]. Early classic features include violaceous, painful, plaque-like subcutaneous nodules, indurations or livedo reticularis [10]. As the disease progresses and vascular thrombosis worsens, lesions become ischemic and necrotic ulcers with eschars (Fig. 2). Lesions involving central body areas with high adiposity are more likely to have classic appearances with eschars compared with those with acral areas such as fingers or toes. Atypical lesions include papules, erythema resembling cellulitis and hemorrhagic crust with erosions.

Diagnosis of calciphylaxis is primarily based on clinical context and physical examination findings. A skin biopsy may be unnecessary, especially among ESKD patients with typical lesions. However, a skin biopsy may be warranted to confirm the diagnosis when the diagnosis is uncertain, as in cases with atypical lesions or typical calciphylaxis lesions without a history of kidney disease. We recommend performing a punch or telescoping biopsy technique by an experienced dermatologist or surgeon, given the risk of provoking new, nonhealing ulcers and secondary infections [11]. The biopsy should be taken from the peripheral, non-necrotic region. Skin biopsy is contraindicated in acral, penile or suspected infected areas [4].

Histologic features of calciphylaxis include calcification, fibrointimal hyperplasia and microvascular thrombosis of the small vessels supplying subcutaneous adipose tissue and dermis. Due to the subtlety of the findings, special staining techniques such as the von Kossa or Alizarin Red stains are recommended to highlight calcium and phosphate deposits [12].

## TIP 3. PAY ATTENTION TO THE CLINICAL CONTEXT, LOCATION OF THE LESIONS AND DIAGNOSTIC TESTS FOR MIMICS OF CALCIPHYLAXIS TO HELP DIFFERENTIATE CALCIPHYLAXIS FROM OTHER CONDITIONS THAT PRESENT SIMILAR TO CALCIPHYLAXIS

**Figure 3:**
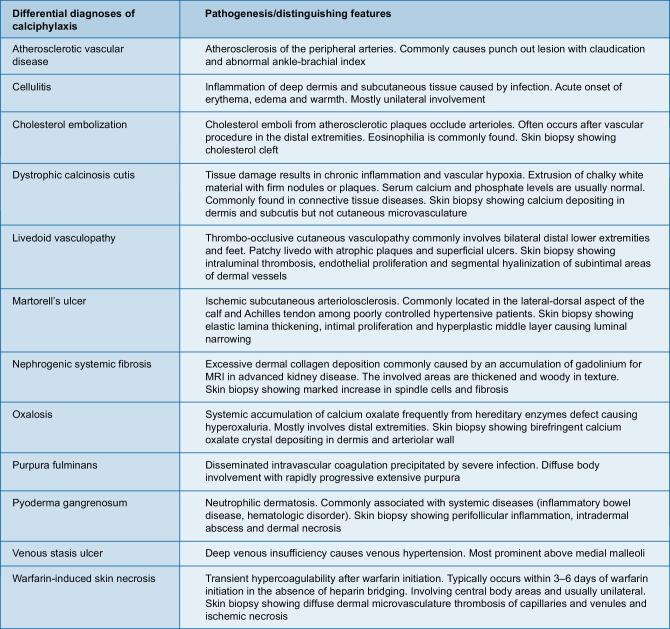
Differential diagnoses of calciphylaxis.

Many conditions can present with features similar to calciphylaxis. Clinical context, location of the lesions and the use of select diagnostic tests help distinguish calciphylaxis from its mimics, as outlined in Fig. 3 [4]. Patients with calciphylaxis normally do not have signs of vasculitis, such as palpable purpura. Additionally, most patients with calciphylaxis have intact peripheral pulses, bilateral necrosis and involvement of the upper extremity, which are different from atherosclerotic disease [13].

Genetic and regional background may contribute to calciphylaxis susceptibility. Previous study demonstrated an unusually high calciphylaxis incidence and poorer survival outcomes among Māori patients undergoing dialysis in Australia and New Zealand, suggesting a potential genetic predisposition [2]. Polymorphisms in key genes involved in mineral metabolism and vascular calcification, including *NT5E* encoding CD73, vitamin D receptor and *FGF23* genes, have been associated with an increased risk of calciphylaxis [13]. Given these genetic and geographic influences, obtaining a detailed regional background and family history can provide valuable diagnostic insights when evaluating suspected cases of calciphylaxis.

## TIP 4. DO NOT ROUTINELY ORDER IMAGING STUDIES FOR THE DIAGNOSIS OF CALCIPHYLAXIS

The role of imaging studies in calciphylaxis diagnosis remains unclear. Plain radiographic findings of small vessel calcification and a netlike pattern of calcification have been reported to be associated with calciphylaxis. A small retrospective study of 29 patients with calciphylaxis demonstrated a 90% specificity of a netlike pattern of calcification of the subcutaneous soft tissue [14]. Another study of 32 patients with calciphylaxis reported the utility of the radiomics-based method using computed tomography scan, with a sensitivity of 89% and specificity of 80% for diagnosing calciphylaxis [15]. Imaging studies may assist in diagnosing calciphylaxis in cases where a skin biopsy is inconclusive or contraindicated [4].

Bone scintigraphy, such as a three-phase technetium 99m methylene diphosphate bone scan, has been reported to be highly sensitive and specific for calciphylaxis diagnosis and may help with early diagnosis before ulcer development. However, variability in interpretation among radiologists is considerable, as there is no standardized grading system for bone scans in calciphylaxis [16]. Given this limitation, we suggest that bone scintigraphy may be used to monitor response to treatment, as demonstrated by a diffuse decrease in skin uptake, instead of assisting in the diagnosing calciphylaxis [17].

## TIP 5. A MULTIDISCIPLINARY APPROACH IS SUGGESTED IN CALCIPHYLAXIS MANAGEMENT

Managing calciphylaxis requires a multidisciplinary approach involving expertise from dermatology, nephrology, nutrition, pain and palliative medicine, plastic surgery, wound care and therapy [18]. The severity of the lesions, along with the need for more intensive dialysis, can contribute to significant psychological distress and depression, necessitating the involvement of mental health professionals to provide psychosocial support, coping strategies and counseling.

Early involvement of the wound care team is essential for selecting appropriate dressings, using chemical debridement agents, and applying negative pressure wound therapy. Surgical debridement by an experienced wound surgeon or plastic surgeon is suggested for noticeably infected or necrotic wounds without signs of granulation tissue. Serial wound debridement should be combined with negative pressure therapy and followed by a split-thickness skin graft [19].

Nutritional optimization plays a central role in calciphylaxis management, particularly in addressing protein-energy malnutrition and supporting wound healing [20]. A normalized protein catabolic rate of at least 1.2 g/kg/day is recommended [21, 22]. Enteral nutrition is preferred when tolerated, while intradialytic parenteral nutrition may be considered for patients with persistent weight loss or severe hypoalbuminemia despite oral dietary supplementation [23]. Most calciphylaxis patients experience severe hypoalbuminemia, which may contribute to disease progression, as albumin—similar to fetuin-A—acts as an endogenous inhibitor of vascular calcification [24]. However, the role of intravenous (IV) albumin infusion in correcting hypoalbuminemia in calciphylaxis remains unclear. While some case reports suggest potential benefits, others have linked albumin transfusions to calciphylaxis onset [25]. Given this uncertainty, we do not recommend routine IV albumin infusion for hypoalbuminemia correction in calciphylaxis patients.

Multimodal pain control with analgesics with different mechanisms of action, biofeedback and relaxation should be emphasized to minimize opioid use and its side effects. Regional anesthesia, such as lumbar sympathetic block, showed success in treating refractory pain [26]. Early involvement in palliative care is crucial, not only for symptom management like pain, pruritus, and loss of sleep or appetite, but also for improving the quality of end-of-life care [27].

## TIP 6. MANAGEMENT OF CKD–MINERAL BONE DISORDER IS AN IMPORTANT CONSIDERATION IN MANAGING PATIENTS WITH CALCIPHYLAXIS

Controlling serum calcium, phosphate and parathyroid hormone (PTH) levels is pivotal for managing calciphylaxis. The goal is to maintain serum phosphate within a normal range and avoid hypercalcemia [20]. Non-calcium-containing phosphate binders such as sevelamer or lanthanum carbonate are recommended to help achieve the goal. Calcium-based phosphate binders are generally avoided as they increase the risk of vascular calcifications [21]. Moreover, calcium supplements and vitamin D analogs should be discontinued.

Currently, there is no strong evidence to support the use of cinacalcet or surgical parathyroidectomy in patients with calciphylaxis. However, we recommend maintaining serum PTH levels between 150 and 300 pg/mL. In patients with calciphylaxis who have serum PTH levels >300 pg/mL, calcimimetics such as cinacalcet should be initiated [22]. The dose of cinacalcet should be titrated to maintain PTH within the desired range. A *post hoc* analysis of the EVOLVE trial demonstrated a 69%–75% reduction in calciphylaxis risk among hemodialysis patients with moderate to severe hyperparathyroidism treated with cinacalcet [23]. Over suppression of PTH to <100 pg/mL should also be avoided as this increases the risk of adynamic bone disease, predisposing patients to vascular calcification and calciphylaxis [24]. Previous study reported that 70% of patients with calciphylaxis exhibit histological evidence of adynamic bone disease [25]. This underscores the potential risk of over suppressing bone remodeling to maintain recommended PTH levels. Incorporating bone turnover markers such as bone-specific alkaline phosphatase, procollagen type 1 N-terminal propeptide (P1NP) and tartrate-resistant acid phosphatase 5b (TRAP5b), or performing a bone biopsy when feasible, could help refine treatment strategies and prevent the inadvertent development of adynamic bone disease [28]. A recent meta-analysis of observational studies demonstrated that cinacalcet use was not associated with a lower risk of mortality, wound deterioration or amputation. However, surgical parathyroidectomy was associated with a lower risk of wound deterioration but not mortality [29].

## TIP 7. DIALYSIS TREATMENTS SHOULD REACH ADEQUACY TARGETS IN DIALYSIS-DEPENDENT PATIENTS WHO DEVELOP CALCIPHYLAXIS

Optimizing the dialysis prescription to meet the National Kidney Foundation-Kidney Disease Outcomes Quality Initiative (NKF-KDOQI) guidelines for dialysis adequacy is recommended for managing calciphylaxis [30]. There is no evidence to support intensifying dialysis beyond the goals of dialysis adequacy solely for calciphylaxis. However, increasing the duration or frequency of hemodialysis may be considered for addressing bone and mineral abnormalities, including refractory hyperphosphatemia, hypercalcemia and hyperparathyroidism, despite optimal dietary restrictions and pharmacologic interventions. We recommend no specific kidney replacement modality for calciphylaxis patients. Calciphylaxis patients should be dialyzed against a low calcium bath to avoid hypercalcemia. Moreover, post-dialysis alkalosis should be avoided as alkalinization potentiates vascular calcium deposition particularly in uremic milieu [31]. We recommend dialysate solutions containing 2.5 mEq/L (1.25 mmol/L) of calcium to mimic normal ionized calcium level in plasma of 5 mg/dL and no more than 35 mEq/L of bicarbonate.

Conversion from peritoneal dialysis to hemodialysis is not routinely warranted unless there are other indications to transition to hemodialysis. Intensifying the prescription of hemodialysis beyond standard adequacy parameters may be considered for patients who have persistent mineral bone disease derangement despite dietary and pharmacological treatments. Case reports have demonstrated complete healing of a large ulcer among calciphylaxis patients on home hemodialysis six times weekly [32, 33]. Limited evidence suggests that kidney transplantation may result in a full resolution of calciphylaxis [34].

## TIP 8. DO NOT CONTINUE WARFARIN IN CALCIPHYLAXIS PATIENTS WHEN POSSIBLE

Warfarin, a vitamin K antagonist, is associated with a higher risk of calciphylaxis [5]. Vitamin K plays a pivotal role in activating γ-carboxylated matrix glutamate protein (MGP) and γ-carboxylated glutamate-rich protein (GRP), which are circulating inhibitors of calcification. Therefore, warfarin use and nutritional vitamin K deficiency can impair these proteins’ functions, promoting the development and progression of calciphylaxis [35].

Although vitamin K supplementation has a theoretical benefit in calciphylaxis, we do not routinely recommend it due to insufficient data on its efficacy and safety. While there is no definitive evidence that correcting vitamin K deficiency can fully resolve calciphylaxis or vascular calcification, some case series have reported complete wound healing and ulcer closure with vitamin K1 (phylloquinone) therapy [36, 37]. Preliminary results from the VitK-CUA trial suggest that vitamin K1 may help alleviate pain and reduce total lesion surface area in calciphylaxis, even in the absence of cinacalcet or intralesional sodium thiosulfate use [38]. Furthermore, findings from the VitaVasK trial indicate a potential reduction in cardiovascular calcification in hemodialysis patients receiving vitamin K supplementation [39]. Several ongoing clinical trials continue to investigate the effects of vitamin K on arterial stiffness and aortic calcification [40, 41]. More robust evidence is needed to establish its role in managing vascular calcification and calciphylaxis.

For patients requiring anticoagulation, transitioning away from warfarin is recommended whenever possible. In those taking warfarin for nonvalvular atrial fibrillation or deep venous thrombosis/pulmonary embolism, a direct oral anticoagulant such as apixaban is preferred, particularly in patients with an estimated glomerular filtration rate <30 mL/min/1.73 m^2^ or ESKD [42]. However, for patients on warfarin due to antiphospholipid antibody syndrome or a mechanical prosthetic heart valve, switching to low-molecular-weight heparin should be considered as an alternative [43].

## TIP 9. A TIME-LIMITED TRIAL OF TREATMENT WITH SODIUM THIOSULFATE IS SUGGESTED

Sodium thiosulfate (STS) is an antioxidant and vasodilatory agent that may also chelate calcium salts and reduce intra- and extravascular calcification [44]. These properties attenuate the ability of adipocytes to induce calcification of the vascular smooth muscle cells [45]. However, recent meta-analyses of observational studies of STS treatment among calciphylaxis patients showed no differences in survival, improvement in skin lesions of amputation [29, 46]. These analyses are limited in terms of the number of patients included in the meta-analyses and heterogeneity between the patients and study designs included in the meta-analyses. Given ongoing clinical trials and limited options for treatment for calciphylaxis, we still suggest a trial of STS for at least 4 weeks.

IV STS is the most commonly used form to treat calciphylaxis. We initiate with a test dose of 12.5 g and titrate up to 25 g three times a week if patients can tolerate the dose. IV STS is administered during the last 30–60 min of hemodialysis sessions. For patients on peritoneal dialysis who cannot receive IV STS or lack of IV access, intraperitoneal STS of 25 g administered with the longest dwell could be considered [47]. Intralesional STS can be administered among patients who cannot tolerate IV or intraperitoneal (IP) STS with a suggested dose of 1–3 mL of 250 mg/mL intralesional STS injected weekly in clinically active calciphylaxis lesions [48]. The optimal duration of STS therapy remains unknown; however, we typically aim for a treatment period of 3 months. We suggest earlier discontinuation among patients who do not demonstrate a response to STS within the first 4 weeks of therapy, particularly when they are also not tolerating this treatment well.

Side effects of STS include volume overload, anion gap metabolic acidosis, nausea and vomiting, corrected QT interval prolongation, hypocalcemia and peritonitis in association with IP administration of STS. To monitor for these complications and guide therapy adjustments, serum calcium, bicarbonate, anion gap and white cell count should be monitored at least once a week and electrocardiogram every 2 weeks.

## TIP 10. POTENTIAL AND FUTURE THERAPIES OF CALCIPHYLAXIS ARE EMERGING

As our understanding of calciphylaxis evolves, several emerging therapies offer promising avenues for treatment.

### Hexasodium phytate (SNF472)

Hexasodium phytate (SNF472) is a hexasodium salt of myo-inositol hexaphosphate which is a potent inhibitor of vascular calcification. Preclinical studies demonstrated the capability of SNF472 to bind hydroxyapatite and prevent the formation and growth of hydroxyapatite crystals *ex vivo*. Phase 1 and 2 clinical trials showed the safety and potential efficacy of SNF472 in attenuating hydroxyapatite crystallization in hemodialysis patients [49, 50]. Furthermore, SNF472 was shown to significantly diminish the progression of coronary artery calcification and aortic valve calcification among ESKD patients at 52 weeks compared with placebo [51].

A recent randomized, double-blind, phase 3, placebo-controlled trial of hexasodium phytate for the treatment of calciphylaxis (CALCIPHYX) conducted among 71 hemodialysis patients (37 patients assigned to SNF472 and 34 patients assigned to placebo) did not meet its primary efficacy endpoints of 8-item modification of the Bates-Jensen Wound Assessment Tool and pain on visual analogue scale. However, a *post hoc* analysis revealed lower mortality rates and calciphylaxis-related events leading to hospitalization among the SNF472 group compared with placebo [52]. Currently, the thrice-weekly intravenous protocol for SNF472—requiring 2–3 h per infusion in the CALCIPHYX trial—represents a significant logistical hurdle outside the in-center hemodialysis setting. Nevertheless, preliminary animal-model data indicate that subcutaneous SNF472 can achieve therapeutic levels [53], supporting further investigation into alternative administration strategies. Future studies are needed to assess the feasibility of subcutaneous, depot or intraperitoneal formulations, potentially expanding SNF472’s accessibility to peritoneal dialysis and non-dialysis patients.

### Hyperbaric oxygen therapy

Hyperbaric oxygen therapy (HBOT) enhances tissue oxygenation, promoting wound healing, reducing infection risk and alleviating the pain. Previous case reports and case series suggested the early initiation of HBOT, before extensive necrosis develops, may improve wound healing [18, 19]. However, HBOT use is limited due to high cost, restricted access to hyperbaric chamber, logistical challenges related transportation and potential discomfort (e.g. claustrophobia).

### Rheopheresis

Rheopheresis is a double-filtration apheresis technique designed to remove high molecular weight proteins such as α2-macroglobulin and proinflammatory cytokines reducing blood viscosity, improving tissue oxygenation and mitigating systemic inflammation [54]. Previous study of eight severe calciphylaxis patients demonstrated that rheopheresis is a safe therapeutic option for calciphylaxis with notable improvement in wound appearance and size occurring shortly after initiation [26]. While preliminary findings are promising, further controlled trials are needed to define optimal patient selection and long-term benefits.

### INZ-701

Inorganic pyrophosphate (PPi) is a potent inhibitor of ectopic vascular calcification and low PPi is associated with calciphylaxis and worse survival outcomes [27]. Ectonucleotide pyrophosphatase/phosphodiesterase 1 (ENPP1) is an enzyme responsible for PPi generation and strategies aimed at enhancing ENPP1 activity could help restore PPi levels and reduce vascular calcification as well as calciphylaxis. INZ-701 is a fusion protein containing functional ENPP1 enzyme and is currently under clinical investigation to assess its safety, pharmacokinetics and pharmacodynamics in hemodialysis patients. By increasing PPi levels, INZ-701 holds promise as a potential therapy for preventing or treating calciphylaxis.

## CONCLUSION

Calciphylaxis should be suspected in patients with advanced CKD or ESKD who present with painful necrotic skin lesions, particularly in areas of high adiposity. Diagnosis often hinges on clinical presentation, with skin biopsy reserved for atypical lesions or unclear cases. Imaging may be useful for monitoring treatment response or when biopsy is contraindicated or inconclusive. Effective management requires multidisciplinary approach, integrating dermatology, nephrology, wound care, nutrition, pain control and therapy. CKD–mineral bone disorder parameters should be optimized, ensuring dialysis adequacy, and discontinuation of warfarin when feasible. A time-limited trial of sodium thiosulfate is suggested, though outcomes remain variable. Future therapies, such as SNF472, HBOT, rheopheresis and INZ-701, are under investigation and may offer promising avenues for improving outcomes in calciphylaxis care. Continued research and clinical trials are essential to refine treatment strategies and expand therapeutic options for this challenging condition.

## Data Availability

No new data were generated or analyzed in support of this research.
